# Pleural and pulmonary dissemination patterns from gastric adenocarcinoma among patients with treated primary disease in Latin America

**DOI:** 10.3389/fsurg.2022.969397

**Published:** 2022-09-07

**Authors:** Juliana Restrepo, Carlos Andrés Carvajal-Fierro, Helena Facundo, Felipe González, Ana María Ramírez, Rafael Beltran, Ricardo Buitrago, Andrés-Felipe Jimenez, José Carreño, Ricardo Oliveros

**Affiliations:** ^1^ Surgical Oncology Department, National Cancer Institute, Bogotá, Colombia; ^2^ Thoracic Surgery Department, National Cancer Institute, Bogotá, Colombia; ^3^ Gastro-intestinal Surgery Department, National Cancer Institute, Bogotá, Colombia; ^4^ General Surgery, National Cancer Institute, Bogotá, Colombia; ^5^ Epidemiology, National Cancer Institute, Bogotá, Colombia

**Keywords:** stomach neoplasms, lymphatic metastasis, neoplasm metastasis, solitary pulmonary nodule, multiple pulmonary nodules

## Abstract

**Purpose:**

Latin America is one of the regions with the highest incidence of gastric cancer. Even though, there are not reports about the patterns of pleuro-pulmonary metastases in patients with gastric adenocarcinoma treated with curative intent and the prognosis according to each dissemination pattern.

**Material and methods:**

We conducted a retrospective analysis of patients with gastric adenocarcinoma treated with curative intent at the National Cancer Institute (INC) between 2010 and 2017. Demographic variables, variables associated with the primary disease and variables associated with the presence of pleuro-pulmonary opacities and metastases were collected. A univariate and multivariate logistic regression analysis was performed and survival curves were presented using the Kaplan Meier method and compared using the log-rank test. A Cox regression model was performed for multivariate analysis for overall survival.

**Results:**

The study included 450 patients, 51.3% were male and the median age was 63 years. Intestinal adenocarcinoma was the most frequent histological subtype, in 261 cases (58.0%). Gastric cancer initial pathological stage was stage I in 23.3% of the patients, stage II in 19.3% and stage III in 53.6%. During a median follow-up of 31.9 months, 37 (8.2%) patients developed pleuro-pulmonary opacities; among those, 14 (3.1%) met the criteria for pleuro-pulmonary metastases: 6 (1.3%) had lymphangitic metastasis, 4 (0.9%) had a mixed pattern of pleural and lung nodules, 3 (0.7%) had pleural metastasis, and only one (0.2%) had hematogenous metastasis. The median OS was 114.5 months for the entire cohort and 38.2 (95%CI, 19.2–57.2) months for patients with pleuro-pulmonary metastases. Patients with pleural metastasis and lymphangitic carcinomatosis had median survival of 24.3 (95%CI, 0.01–51.0) and 26.4 (95%CI, 18.2–34.7) months, respectively.

**Conclusions:**

incidence of pleuro-pulmonary metastases in patients with gastric adenocarcinoma treated with curative intention was low. In our series, lymphangitic carcinomatosis was the main pattern of dissemination; meanwhile, hematogenous metastasis was rare and patients with pleural carcinomatosis had the lowest median survival.

## Introduction

“GLOBOCAN is an online database that provides global cancer estimates of incidence, mortality and prevalence; for the year 2020 it included 185 countries or territories for 36 cancer types by sex and age group. The data is part of the International Agency for Research on Cancer's Global Cancer Observatory, and is available online at Cancer Today with user-friendly facilities to produce maps and explore visualizations.” In 2020, the GLOBOCAN described that gastric cancer was responsible for more than one million of new cases and estimated 769.000 deaths, making this cancer the fifth and fourth in terms of frequency and mortality, respectively (1, 2). In Colombia, Pardo et al. described in 2018 an age-standardized incidence rate (ASIR) in men of 18.5 per 100.000 population corresponding to second place in frequency and in women of 10.3 per 100.000 population occupying fourth place. Meanwhile, the age-standardized cancer mortality rate (ASMR) in men was 14.2, representing the leading cause of cancer deaths (3). The stage at diagnosis in our country is different from those reported in eastern countries where most patients are diagnosed in early stages of the disease due to screening programs.

The recurrence rate of gastric cancer patients treated with complete surgical resection is between 30% and 50%, generally occurring within the first two years after gastrectomy (4), and distant recurrence is present in more than 50% of cases (5). According to Kong et al. (6), hematogenous pattern of lung metastasis was the main pattern of dissemination with almost 45% of the 193 patients with pleuro-pulmonary metastasis in their study that included 20.187 patients with early to advanced or metastatic gastric cancer in Korea.

Even though, America occupies third place in gastric cancer incidence, after Asia and Europe (7); there are not reports about the patterns of pleuro-pulmonary metastases in these patients. This study aimed to identify pleural and pulmonary dissemination patterns in patients with gastric adenocarcinoma treated with curative intent at National Cancer Institute (INC) in Colombia. Our findings will allow us to define the prognosis according to each dissemination pattern and to understand the disease biology in a more comprehensive way.

## Materials and methods

### Study population

This was an analytic and retrospective cohort study. *Inclusion criteria* were: patients with gastric adenocarcinoma treated with curative intent at INC between January 2010 and July 2017, without history of other cancer except for non-melanoma skin cancer managed with local therapies. *Exclusion criteria* were: patients younger than 18 years, with metastatic disease at diagnosis, with tumors of the esophago-gastric junction (Siewert classification I and II), with positive surgical resection margins, or those with positive peritoneal fluid cytology. Lung and pleural surveillance protocol of gastric cancer patients treated with curative intent included CT-Scan every 6 months for 2 years after surgery and annually thereafter for the next 3 years. Respiratory symptoms at any time of follow-up indicated CT-Scan.

### Statistical analysis

A pre-designed RedCap 7.1.2 © format was used to collect the data. Age and sex were analyzed as demographic variables, and the variables for the primary disease included were location, stage, gastric surgery performed, histologic subtype, and other histologic variables like perineural invasion and linfovascular invasion. Variables associated with the pleural or pulmonary disease progression, such as the type of pleuro-pulmonary compromise, were also analyzed and, when pulmonary nodules were present, the number, radiologic characteristics, size, location, and histology were described. Other variables included were the treatment received by the patient (i.e., adjuvant chemotherapy or palliative chemotherapy) and, in the cases where pulmonary resection was performed; we also assessed the type of surgery. Concomitant metastatic sites were not described in the present study.

The data were analyzed using SPSS (IBM SPSS Statistics for Windows, version 19.0, released 2010, IBM Corp., Armonk, NY, United States). Descriptive statistics were used to report the medians and interquartile range (IQR) of continuous variables (25th and 75th centiles given as the IQR). Number and percentage were used to describe categorical variables. Survival curves were presented using the Kaplan Meier method and compared using the log-rank test. A univariate and multivariate logistic regression analysis was performed to identify the predictors of pleuro-pulmonary opacities and metastases and odds ratio (OR) were reported. A Cox regression model was performed for multivariate analysis for overall survival. *P* ≤ 0.05 was considered statistically significant.

### Survival definitions

*Overall survival* (OS) was defined as the time elapsed in months between surgery with curative intent for gastric cancer and the date of death or the last follow-up.

*Hematogenous metastasis* was defined as solitary or multiple lung nodules on Computed Tomography (CT) scan, in patients who underwent resection and had histopathologic confirmation. Nodules with a benign histopathologic report or stability in the follow-up on the CT-scan were not classified as hematogenous metastases. *Pleural metastasis* was defined as pleural thickening or effusion on CT-scan or chest x-ray with histopathologic confirmation or highly suspicious CT-scan findings. *Lymphangitic metastasis* was defined as interstitial patterns such as thickening or irregularity of interlobular septa and bronchovascular bundles on CT-scan consistent with lymphangitic carcinomatosis. Histopathologic confirmation was not mandatory.

The ethics committee at our institution approved the protocol before collecting the patient's data, Act No. 0023-19.

## Results

We found 450 patients with gastric adenocarcinoma treated with curative intent and negative surgical resection margins; male gender was predominant, with 51.3% of the patients being male and having a median age of 63 years (IQR 53–71). The gastric tumor location was cardial in 62 patients (13.8%) and non-cardial in 386 (85.8%). The type of gastric surgeries performed were subtotal gastrectomy in 216 patients (48.0%) and total gastrectomy in 202 (44.9%), see Table 1. Intestinal adenocarcinoma was the most frequent histological subtype, observed in 261 cases (58.0%), followed by diffuse in 116 cases (25.8%) and mixed in 45 cases (10.0%). Perineural invasion (PNI) of the gastric tumor was reported in 235 cases (52.2%) and lymphovascular invasion (LVI) in 296 (65.8%). Human epidermal growth factor receptor 2 (HER2) status was assessed by immunohistochemistry (IHC) in 190 patients, and overexpression was found in 17 of them (8.9%), see Table 1.

**Table 1:** Demographic, gastric cancer, surgical and follow-up variables.

Variable (*n* = 450)	No (%)/median (IQR)
Age (years)	63 (53–71)
Male	231 (51.3)
**Primary tumor variables**	
Location (missing data: 2)	
Non-cardial	386 (85.8)
Cardial	62 (13.8)
Type of gastric surgery	
Subtotal gastrectomy	216 (48.0)
Total gastrectomy	202 (44.9)
Total gastrectomy with esophageal margin	32 (7.1)
Stage^a^	
I	105 (23.3)
II	87 (19.3)
III	241 (53.6)
Incomplete lymph node count	17 (3.8)
Histologic subtype, (missing data: 28)	
Intestinal	261 (58.0)
Diffuse	116 (25.8)
Mixed	45 (10.0)
Lymphovascular invasion (missing data: 78)	296 (65.8)
Perineural invasion, (missing data: 146)	235 (52.2)
HER 2 positive (missing data: 260)	17 (8.9)
Additional treatments	
Neoadjuvant chemotherapy	34 (7.6)
Adjuvant chemotherapy	298 (66.2)
Palliative chemotherapy	45 (10.0)
Neoadjuvant radiotherapy	14 (3.1)
Adjuvant radiotherapy	209 (46.4)
Palliative radiotherapy	11 (2.4)
Median size of pulmonary nodules (mm.)	8 (3–27)
Patients taken to pulmonary nodule resection	*n* = 8
Type of pulmonary resection	
Wedge resection	6 (75.0)
Anatomic segmental resection	1 (12.5)
Lobectomy	1 (12.5)
Number of nodules resected per patient, (*n*: 8)	
1	4 (50.0)
2	4 (50.0)
Histology of the resected nodules, (*n*: 8)	
Benign nodule	6 (75.0)
Primary pulmonary adenocarcinoma	1 (12.5)
Gastric cancer metastasis	1 (12.5)

^a^ Stage AJCC 2018.

Gastric cancer initial pathological stage was stage III in 241 cases (53.6%), stage II in 87 (19.3%), and stage I in 105 (23.3%). Neoadjuvant and adjuvant treatment with chemotherapy and radiotherapy is summarized in Table 1.

**Figure 1:**
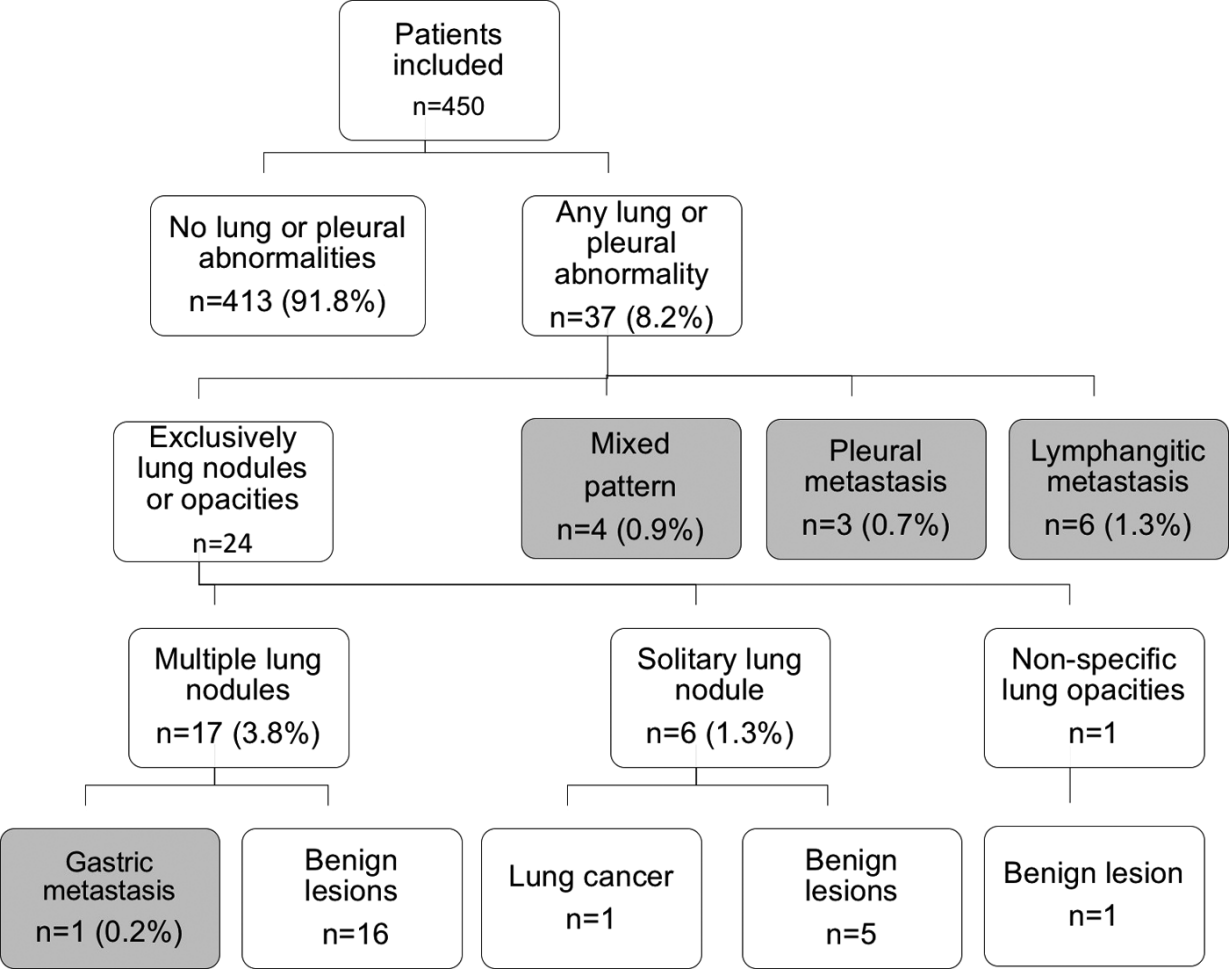
Flow chart of included patients. Grey color in the boxes corresponds to the 14 patients that met the criteria for pleural or pulmonary metastases.

The median follow-up time was 31.9 months (IQR 12.4–57.9 months), being over 36 months in 205 patients (45.6%) and below 6 months in 55 patients (12.2%). While 413 patients (91.8%) did not develop pleuro-pulmonary opacities, these abnormalities were observed in 37 of them (8.2%). We found multiple lung nodules in 17 patients (3.8%), lymphangitic carcinomatosis in 6 (1.3%), solitary lung nodule in 6 (1.3%), mixed pattern of lung nodules and pleural metastases in 4 (0.9%), pleural metastasis in 3 (0.7%) and non-specific lung opacities in one. Among these 37 patients with pleuro-pulmonary opacities, 14 (3.1%) met the criteria for pleuro-pulmonary metastases. 6 (1.3%) had lymphangitic metastasis, 4 (0.9%) had a mixed pattern of pleural and lung nodules, 3 (0.7%) had pleural metastasis, and only one (0.2%) had hematogenous metastasis, see Figure 1. None of the patients with lymphangitic carcinomatosis and with mixed pattern on the CT-scan underwent surgical confirmation, since they met the criteria for metastasis. Pleural metastases were confirmed by histology and the patient with hematogenous metastasis had multiple lung nodules and was confirmed by wedge resection. Among the 23 patients with lung or pleural abnormalities that did not met the criteria for metastases, one had a solitary lung nodule and it's etiology was lung cancer, the other lesions were considered benign by histopathologic confirmation or stability on the CT-scan in the follow-up. Of the 8 resected nodules, one patient had gastric cancer metastases and was a patient with multiple solid pulmonary nodules, the largest being 8 mm in diameter. Another patient had lung cancer and had a spiculated solid pulmonary nodule, 12 mm in diameter. The other 6 benign nodules: 5 solid and one subsolid, with a median diameter of 12.5 mm and three multiple. The size and number of resected pulmonary nodules are described in Table 1.

**Figure 2:**
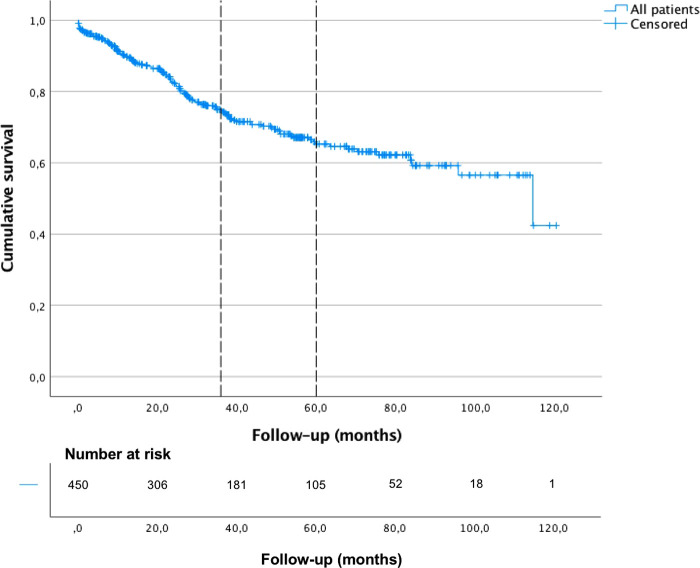
Overall survival of gastric cancer patients treated with curative intent, Kaplan-Meier curve.

**Figure 3:**
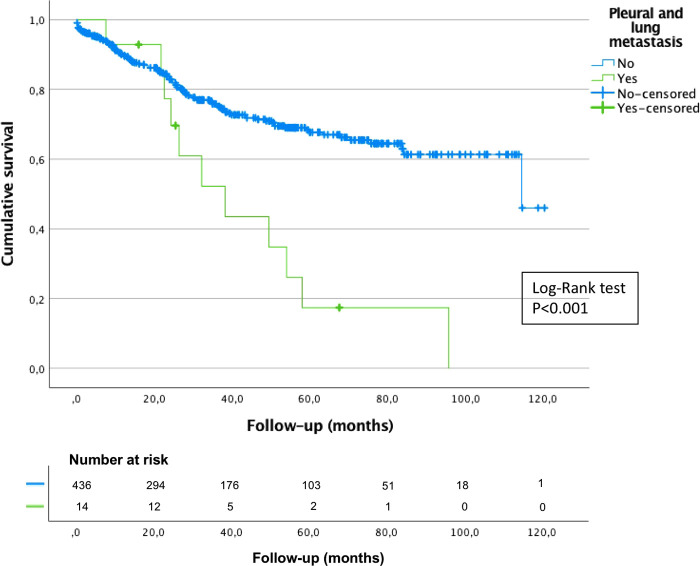
Overall survival of gastric cancer patients treated with curative intent who developed or not pleuro-pulmonary lung metastases, Kaplan-Meier curve with Log-Rank test.

The median OS was 114.5 months (95% CI, 81.6–147.4) for patients with gastric adenocarcinoma treated with curative intent, and their 3-year and 5-year OS was 75% and 65%, respectively, see Figure 2. The median time for developing pleuro-pulmonary metastases after gastric surgery was 22.3 months (IQR 9.5–28.3) and the median survival was 38.2 months (95% CI, 19.2–57.2), 3-year and 5-year OS was 53% and 18% for patients with pleuro-pulmonary metastases, respectively. There was a statistically significant difference between patients who developed pleuro-pulmonary dissemination or not, see Figure 3. On the other hand, the median survival after the diagnosis of pleuro-pulmonary metastases was 1.1 months (IQR 0.1–22.6 months). Patients with pleural carcinomatosis had the lowest median survival, 24.3 months (95% CI, 0.01–51.0); followed by patients with lymphangitic carcinomatosis with a median survival of 26.4 (95% CI, 18.2–34.7) and patients with mixed progression that had a median survival of 54.1 (95% CI, 46.7–61.4). The only patient with hematogenous metastasis to the lung parenchyma survived 32.2 months.

Univariate and multivariate analysis was performed to evaluate predictors of pleuro-pulmonary opacities as well as pleuro-pulmonary metastases. We found that, in the univariate and multivariate analysis, patients who received adjuvant chemotherapy were associated with developing pleuro-pulmonary opacities OR:3.92 [95%CI(1.35–11.33) *P* = 0.022] and OR:6.49 [95%CI(1.32–31.97) *P* = 0.022], respectively; but, this association was not found in the univariate or the multivariate analysis with pleuro-pulmonary metastases, see Tables 2, 3.

**Table 2:** Univariate analysis for predictors of developing pleuro-pulmonary opacities and metastases among patients with gastric adenocarcinoma treated with curative intent.

Variable	Pleuro-pulmonary opacities (*n* = 37)		Pleuro-pulmonary metastases (*n* = 14)	
	OR (95% CI)	*P* value	OR (95% CI)	*P* value
Gender				
Male	0.54 (0.27–1.10)	0.089	0.58 (0.19–1.74)	0.330
Location				
Cardial	0.97 (0.36–2.59)	0.952	1.04 (0.23–4.76)	0.961
Stage of primary disease				
I	Ref		Ref	
II	2.21 (0.62–7.81)	0.219	5.01 (0.55–45.70)	0.153
III	2.41 (0.81–7.20)	0.115	3.11 (0.38–25.61)	0.291
Adjuvant chemotherapy	3.92 (1.35–11.33)	**0.012**	1.74 (0.48–6.33)	0.402

OR: Odds ratio, CI: confidence interval.

**Table 3:** Multivariate analysis for predictors of developing pleuro-pulmonary opacities and metastases among patients with gastric adenocarcinoma treated with curative intent.

Variable	Pleuro-pulmonary opacities (n=37)		Pleuro-pulmonary metastases (n=14)	
	OR (95% CI)	*P* value	OR (95% CI)	*P* value
Gender				
Male	0.66 (0.31–1.44)	0.301	0.72 (0.22–2.36)	0.588
Location				
Cardial	0.92 (0.30–2.84)	0.891	0.82 (1.17–3.98)	0.805
Stage of primary disease				
I	Ref		Ref	
II	1.15 (0.25–5.22)	0.858	3.84 (0.34–43.76)	0.278
III	1.18 (0.30–4.70)	0.814	2.30 (0.22–23.88)	0.486
Adjuvant chemotherapy	6.49 (1.32–31.97)	**0.022**	1.54 (0.28–8.53)	0.621

OR, Odds ratio; CI, confidence interval.

A Cox regression model was also performed for OS among the entire cohort. We found that cardial location HR: 2.26 [95%CI (1.24–4.11) *P* = 0.008] and diffuse histology HR: 2.12 [95%CI (1.19–3.79) *P* = 0.011], were independent factors of worse OS. Meanwhile, adjuvant chemotherapy and stage I of the primary disease were independent factors for better OS HR: 0.24 [95%CI (0.13–0.42) *P* < 0.001] and HR: 0.035 [95%CI (0.006–0.213) *P* < 0.001], respectively, see Table 4.

**Table 4:** Cox regression model for OS among the entire cohort.

Variable	Pleuro-pulmonary opacities (*n* = 37)	
	HR (95% CI)	*P* value
Age (years)		
≥60	0.95 (0.55–1.63)	0.849
Gender		
Male	0.89 (0.55–1.42)	0.621
Location		
Cardial	2.26 (1.24–4.11)	**0** **.** **008**
Stage of primary disease		
I	0.035 (0.006–0.213)	**<0** **.** **001**
II	0.723 (0.33–1.59)	0.421
III	Ref.	
Histology subtype		
Intestinal	Ref	
Diffuse	2.12 (1.19–3.79)	**0** **.** **011**
Mixed	1.13 (0.53–2.42)	0.744
Lymphovascular invasion	0.62 (0.14–2.65)	0.515
Perineural invasion	1.92 (0.60–6.13)	0.273
Adjuvant chemotherapy	0.24 (0.13–0.42)	**<0** **.** **001**

HR, Hazard ratio.

## Discussion

In this retrospective cohort study, the percentage of patients who developed pleuro-pulmonary opacities was 8.2% during a median follow-up of 31.9 months. However, of these patients, only 3.1% had pleuro-pulmonary metastases, according to highly suspicious findings in the images or histopathologic confirmation. Most of these patients had lymphangitic carcinomatosis and pleural metastases. Only one patient with multiple lung nodules had metastasis from gastric adenocarcinoma, and none of the patients with solitary nodules had hematogenous metastases; meaning that only 0.2% of this cohort had metastatic nodules in the lung parenchyma. These results are consistent with the very low possibility that the etiology of new pulmonary nodules in the follow-up of patients with gastric cancer with previous curative management be metastases. As expected, patients with pleuro-pulmonary metastases of the disease had lower OS.

In the univariate and the multivariate analysis, adjuvant chemotherapy was a predictor factor for developing pleuro-pulmonary opacities. Drug-induced interstitial pneumonia is one of the many adverse reactions induced by anticancer regimens. FOLFOX is one of the regimens related to this reaction, includes folinic acid, fluorouracil and oxaliplatin, and is commonly used to treat gastric and colorectal cancer. But, manifestations of the drug-induced interstitial pneumonia are unspecific and its diagnosis is usually difficult (8).

In 2020 the highest incidence rates were described in Eastern Asia followed by Eastern Europe and South America whereas the rates in Northern America and Northern Europe were generally low and were equivalent to those seen across the African regions (1). Incidence rates have reached 37.4 per 100.000 population in countries in Eastern Asia, such as China, Korea and Japan (9). In Latin America, high-risk pockets are reported in the Andes Mountains, with incidence rates between 20 and 30 for countries located in this zone, such as Chile and Colombia (1, 10). The incidence rates in Colombia vary with high-incidence areas located at high altitudes in the Andes Mountains and low-incidence areas on the coasts and valleys (3).

Tumor location in gastric cancer patients is different between Western and Eastern countries; tumors located in the proximal third are more frequent in western countries and this is associated with more advanced stage at presentation and worse survival (9, 11). Although Colombia has a high gastric cancer incidence in some territories, most patients are diagnosed at an advanced stage and their cancer's biological behavior is closer to that of western countries. In this cohort, more than half of our patients were diagnosed with a stage III disease; 25.8% had diffuse histology, and 13.8% were located in the cardias. Considering our findings, we believe that gastric cancer in western countries, including those from Latin America, has different biological behavior and therefore has different metastasis patterns compared to those reported in Eastern countries.

Distant metastases do not usually reach the lungs and pleura, occurring in 0.3% to 6% of patients treated with curative intent (4). This proportion was 3.1% in our series, but the proportion of patients with metastatic hematogenous seeding to the lung parenchyma was low, 0.22%. In United States, a multi-institutional analysis (12), including 817 patients with gastric cancer treated with curative intent showed that the most common sites of distant recurrence were peritoneum, followed by liver, lung, and bone. The median recurrence-free survival was 10.8 months, and the median time for lung recurrence was 10.1 months. They found several factors related to recurrence, including age, lesion size, histologic type, number of lymph node metastasis, and the presence of lymphovascular invasion or perineural invasion. The median survival after recurrence was five months, and only 2% developed pleuro-pulmonary metastases. In the present study, we found no predictors of progression of gastric adenocarcinoma to the lung or pleura. But in the multivariate analysis among the entire cohort, we found that cardial location and diffuse histology were independent factors of worse OS. Meanwhile, adjuvant chemotherapy and stage I of the primary disease were independent factors for better OS.

Survival after gastric cancer recurrence is low. Even with chemotherapy, the median survival ranges between 6 and 13 months (13). The Memorial Sloan-Kettering Cancer Center study (5), representing a western cohort, described three recurrence patterns of adenocarcinoma: locoregional, peritoneal, and distant or systemic. From 1,172 patients who underwent R0 resection, 42.3% had a recurrence. Most recurrences (79%) occurred in the first two years, and disease recurrence was rare after four years. Locoregional recurrences were present in 54%, peritoneal recurrence in 29%, and distant recurrences in 51%. Only 39 patients had recurrences to the lung parenchyma, representing 3.3% of the entire cohort, similar to our study. The median time from recurrence to death was six months, 70% were dead within one year, and the specific recurrence pattern had no significant effect on the time to death.

The cohort study reported by Kong et al. in 2012 (6), representing an eastern cohort, reported the most extensive series to date; they reviewed 20.187 gastric cancer patients and identified 193 (0.96%) as having metastases to the lung parenchyma, pleura, or lymphangitic metastatic lesions. Even though, the incidence of pleuro-pulmonary metastasis was 3.1% in our cohort, higher than that described by Kong et al. they found that the most frequent pattern of lung metastasis was hematogenous metastasis (44.5%) followed by pleural (24.4%), lymphangitic (18.7%) and mixed (12.6%) metastases. In our series, the main pattern of pleuro-pulmonary metastases was lymphangitic metastasis present in 42.9% of the patients, followed by a mixed pattern in 28.6%, pleural metastasis in 21.4% and only one patient (0.2%) had hematogenous metastasis. They also found that young age was associated with the lymphangitic spread, and the diffuse type was associated with pleural seeding. This cohort is different from ours because it was not restricted to patients treated with curative intent. Finally, Kong et al. described that 80% of the patients with lung metastases of gastric cancer had concomitant metastases of other organs, especially the peritoneum and liver and they found that the median survival after diagnosing pleuro-pulmonary metastases in gastric cancer patients was four months, and 5-year survival was only 2%–4%. In our series, we did not describe the presence of concomitant metastases of other organs and the median survival after diagnosis of pleuro-pulmonary metastases was 1.1 months.

Aurello et al. (13) described that the median time to recurrence was 14 to 29 months after surgery, similar to what we found in our series, where the median time for developing pleuro-pulmonary metastases after gastric surgery was 22.3 months.

Lung metastasectomy has been proposed for several solid tumors, however, little is known about the resection of gastric cancer lung metastases (4). There are many series and case reports describing lung metastasectomy, especially in Asian countries (4, 14–16). In 2010, Kemp et al. (14) addressed this question with a review of the articles on this subject published from 1975 to 2008. They reported 21 studies, including 43 patients with OS or 39 months and five-year survival of 33% after lung metastasectomy and a median survival time of 29 months. Aurello et al. (4) performed a systematic review in 2016 that included 44 patients who underwent lung resection for gastric cancer metastasis between 1998 and 2013; after lung resection, recurrence occurred in 21 patients, and six patients were disease-free at the last follow-up; median OS after gastric resection 45 months, suggesting that lung metastasectomy could improve the survival of patients with lung metastasis from gastric cancer when compared to patients treated with palliative chemotherapy. Shiono et al. (15) reported a 5-year OS of 28% for patients with gastric cancer treated with pulmonary metastasectomy and a median survival time of 29 months. The study by Iijima et al. (16) reported ten patients who underwent pulmonary metastasectomy for gastric cancer in Japan; their primary disease was controlled, they had no other extrapulmonary metastases, and the pulmonary metastases were limited to one lung; the overall 3-year survival was 30%, and the median survival time following pulmonary resection was 26.8 months. In our study, the only patient who had lung metastasectomy survived for 32 months after gastrectomy and 12 months after pulmonary resection. We cannot make any conclusions with one patient, but the median survival after lung metastasis diagnosis and treatment was higher compared with patients with unresected pleuro-pulmonary compromise. The role of pulmonary metastasectomy in gastric cancer needs further studies, and it may be proposed only for selected cases.

Even though, thoracic staging with CT-scan for gastric adenocarcinoma is still controversial (17), we believe it is necessary. In patients with pleural or lymphangitic carcinomatosis, CT-scan findings are usually very suggestive of metastatic compromise. However, in the case of lung nodules, the incidence of metastatic lesions was very low and although this study looked at the development of pulmonary metastases after gastric cancer resection, not at diagnosis; these results may be a starting point for further studies in patients with pulmonary nodules at the time of diagnosis that may change the approach during gastric cancer staging and avoid unnecessary lung resections.

There are several limitations in this study. It was a retrospective analysis of a single institution and it did not describe which patients had concomitant metastatic sites other than the lung and pleura. Details and regimens of chemotherapy and radiotherapy regimens administered in the neoadjuvant, adjuvant, and palliative setting, were also not described. Due to the small number of patients with pleuro-pulmonary metastases, it was not possible to find differences between the survival of each of the patients who presented these dissemination patterns. However, this is the first study that describes the patterns of pleuro-pulmonary metastases in gastric cancer patients treated with curative intent in Latin-America.

## Conclusions

Incidence of pleural or pulmonary metastases in patients with gastric adenocarcinoma treated with curative intent was low. In our series, lymphangitic metastasis was the main pattern of dissemination, meanwhile lung nodules with histopathologic confirmation of hematogenous metastasis were rare. Additionally, patients with pleural carcinomatosis had the lowest median survival, followed by patients with lymphangitic carcinomatosis.

## Data Availability

The raw data supporting the conclusions of this article will be made available by the authors, without undue reservation.

## Supplementary material

Supplementary Figure 1 Overall survival of patients according to metastases pattern, Kaplan-Meier curve with Log-Rank test.
