# Supplementary figures and images for: Pleural and pulmonary dissemination patterns from gastric adenocarcinoma among patients with treated primary disease in Latin America
Source: Front Surg. 2022 Sep 7;9:969397. doi: 10.3389/fsurg.2022.969397 (PMC9489939; doi:10.3389/fsurg.2022.969397)

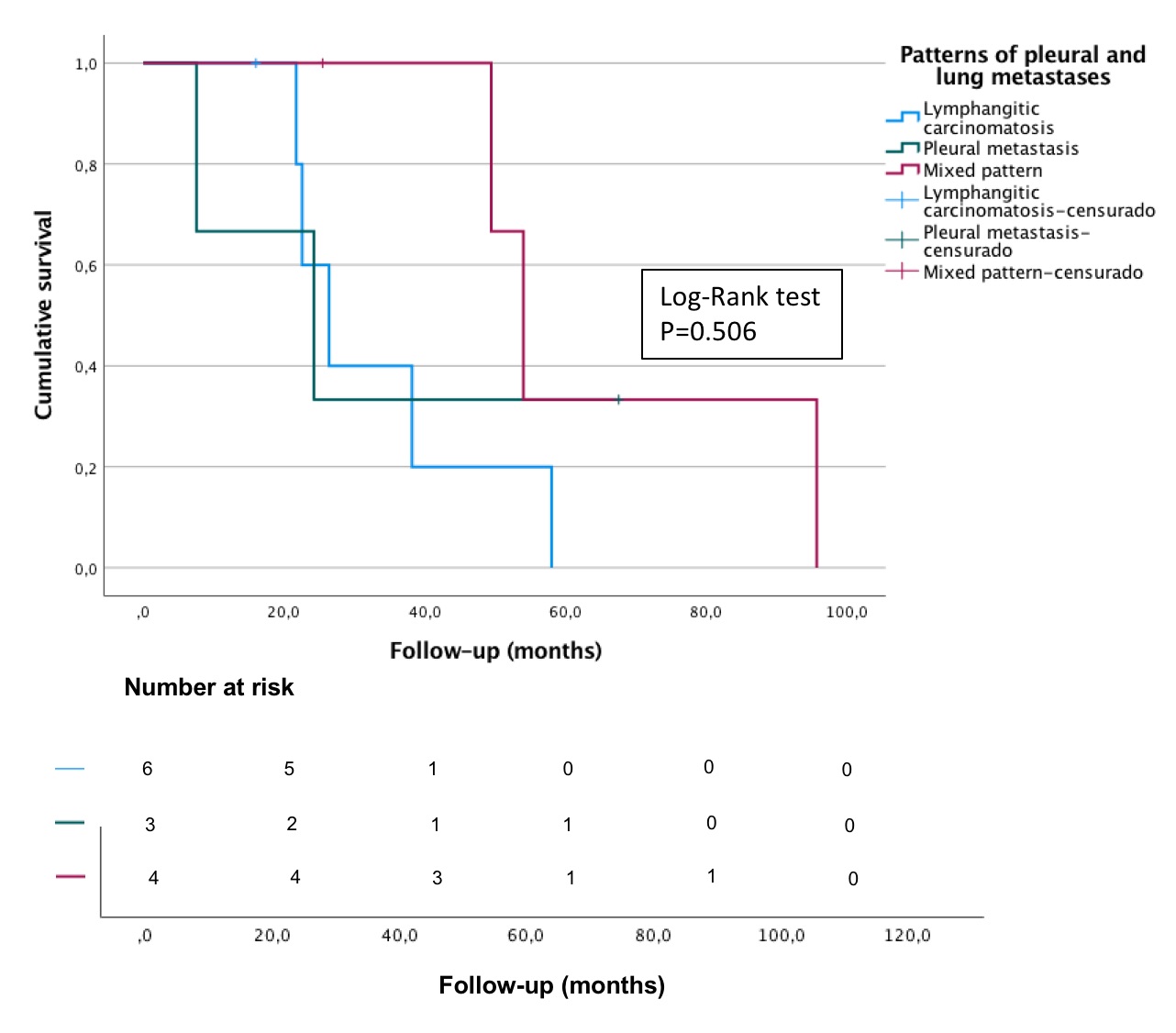

Supplement: Supplementary Figure 1 — Overall survival of patients according to metastases pattern, Kaplan-Meier curve with Log-Rank test. [file Image_1_v1.jpeg]
